# Awake Surgery for Left Posterior Insular Low-Grade Glioma Through the Parietorolandic Operculum: The Need to Preserve the Functional Connectivity. A Case Series

**DOI:** 10.3389/fsurg.2021.824003

**Published:** 2022-01-13

**Authors:** Hugues Duffau

**Affiliations:** ^1^Department of Neurosurgery, Gui de Chauliac Hospital, Montpellier University Medical Center, Montpellier, France; ^2^Team “Plasticity of Central Nervous System, Stem Cells and Glial Tumors, ” National Institute for Health and Medical Research (INSERM), U1191 Laboratory, Institute of Functional Genomics, University of Montpellier, Montpellier, France

**Keywords:** intraoperative mapping, left insula, low-grade glioma, transcortical approach, awake surgery

## Abstract

**Objective:** Surgical approach to low-grade glioma (LGG) involving the posterior insula is challenging, especially in the left hemisphere, with a high risk of sensorimotor, language, or visual deterioration. In this study, a case series of 5 right-handed patients harboring a left posterior insular LGG is reported, by detailing a transcorticosubcortical approach.

**Method:** The five surgeries were achieved in awake patients using cortical and axonal electrostimulation mapping. The glioma was removed through the left rolandic and/or parietal opercula, with preservation of the subcortical connectivity.

**Results:** The cortical mapping was positive in the five patients, enabling the selection of an optimal transcortical approach, *via* the anterolateral supramarginal gyrus in four patients and/or *via* the lateral retrocentral gyrus in three cases (plus through the left superior temporal gyrus in one case). Moreover, the white matter tracts were identified in all cases, i.e., the lateral part of the superior longitudinal fasciculus (five cases), the arcuate fasciculus (four cases), the thalamocortical somatosensory pathways (four cases), the motor pathway (one case), the semantic pathway (three cases), and the optic tract (one case). Complete resection of the LGG was achieved in two patients and near-total resection in three patients. There were no postoperative permanent sensorimotor, language, or visual deficits.

**Conclusion:** A transcortical approach through the parietorolandic operculum in awake patients represents safe and effective access to the left posterior insular LGG. Detection and preservation of the functional connectivity using direct electrostimulation of the white matter bundles are needed in this cross-road brain region to prevent otherwise predictable postsurgical impairments.

## Introduction

Although surgery of insular gliomas was reputed for a long time to be very challenging, there is currently a growing number of series demonstrating a significant improvement of functional and oncological outcomes—see recent reviews on this topic (1–3). Such better results have in particular been correlated to the use of awake surgery with intraoperative electrical mapping [for a meta-analysis, see (4)] and to transopercular approach, which allows a wider surgical exposure of the insula and a decrease of vascular injuries at the level of the Sylvian arteries (5–7). However, surgical access remains complex for gliomas within the left posterior insula (8), explaining why a greater amount of postoperative residue was observed in this location, especially when the tumor involves zone II according to the Berger-Sanai classification system (9). Furthermore, identification of the eloquent subcortical pathways is critical in this cross-road brain region to avoid postoperative severe permanent deteriorations (10).

Here, this case series details a transcortical approach to reach the left posterior insular low-grade glioma (LGG) through the parietorolandic operculum in five patients operated on with awake mapping. Beyond the access to the insula selected according to the results of the individual cortical mapping, the purpose is to emphasize the need to preserve the neural connectivity, thanks to the detection of the white matter tracts by means of subcortical electrostimulation, these pathways serve as deep functional boundaries of the resection.

## Method

### Patients

This is a consecutive case series of patients with an LGG involving the left posterior insula and who underwent awake surgery between October 2015 and March 2021.

Written signed consent was obtained from all patients before surgery. Ethics Committee approval was not required because of the descriptive nature of the study.

Pre- and post-operative functional outcome was assessed systematically *via* neurological examination. All patients underwent preoperative MRI, then postoperative MRI within 24 h postsurgery, at 3-month and 6-month intervals thereafter.

### Surgical Procedure

All the patients underwent awake resection with cortical and subcortical direct electrical stimulation (DES) as extensively described elsewhere (10, 11). Briefly, patients underwent anesthesia induction and placement of a laryngeal mask airway. A local anesthetic was applied, and a left craniotomy was performed. Patients were then woken up and the airway was removed to enable full participation in functional mapping. After durotomy, tumor, sulcal, and gyral boundaries were delineated with intrasurgical ultrasonography to confirm localization. No other technical adjunct was used in the operative theater (no neuronavigation, no functional MRI, no tractography, and no intraoperative MRI).

Direct electrical stimulation (DES) was achieved by means of a bipolar stimulator with tips spaced 5-mm apart. A biphasic current with pulse frequency 60 Hz, single pulse phase duration 1 ms, amplitude 2–3 mA, and stimulation duration of 4 s was applied. In all cases, initial sensorimotor and speech mapping (counting) was achieved to identify negative motor and primary sensorimotor sites, and speech regions (ventral premotor cortex). Then, patients have been asked to complete a dual naming task (eventually with the presentation of two objects situated diagonally on the computer screen to preserve the visual field) combined with a contralateral upper limb movement (12). Cortical areas inducing sensorimotor responses, language disturbances (speech arrest, phonological or semantic paraphasia, and anomia), and/or visual troubles during DES were marked by sterile tags.

Glioma removal then proceeded by first achieving a safe corticectomy through the lateral part of the retrocentral gyrus (lRCG) and/or through the anterolateral part of the anterior supramarginal gyrus (alSMG) according to the results of the cortical mapping. The lateral part of the superior longitudinal fasciculus (lSLF), critical for articulatory processes (13), was then identified and served as the upper limit of the opercular resection. The surface of the insula was subsequently exposed in the depth using a subpial dissection technique. The next step consisted of lifting the circular sulcus of the insula, allowing to provide access to the tumor involving the posterior part of the insular lobe, and removing it by preserving the Sylvian branches (since protected by the pia-matter) (10). Finally, in the white matter underneath the insula, tumor removal and axonal DES mapping were alternated to detect the eloquent pathways which represented the deep functional boundaries of the resection while the patient continued to perform multitasking (movement, language, and/or visual tasks). The goal was to identify and preserve the arcuate fasciculus (AF) (which generates phonological disorders during DES), the inferior fronto-occipital fasciculus (IFOF) (which induces semantics disturbances during DES), the somatosensory thalamocortical pathway (TCP) (which elicits dysesthesia or pain during DES), the pyramidal pathway (PT) (which evokes involuntary movement during DES), and/or the optic radiation (OR) (which causes visual disorders during DES). Resections were thus pursued until critical neural networks were encountered around the whole surgical cavity, with no margin around eloquent structures, to optimize the extent of resection (EOR) (14).

Photographs of cortical and subcortical responses (indicated by sterile tags) were taken before and after resection. Each DES site was manually plotted on the three-month postoperative MRI. Replacement of stimulation point was done by using intrasurgical photos (enabling to accurately identify the location of functional areas marked with sterile numbered tags), written surgical reports, cortical (e.g., gyri, sulci, and vessels) and subcortical (e.g., edges of the resection cavity) landmarks identifiable on both the postsurgical MRI and the intraoperative photographs. This method has been successfully used and validated in the previous series (15–17).

## Results

**Figure 1:**
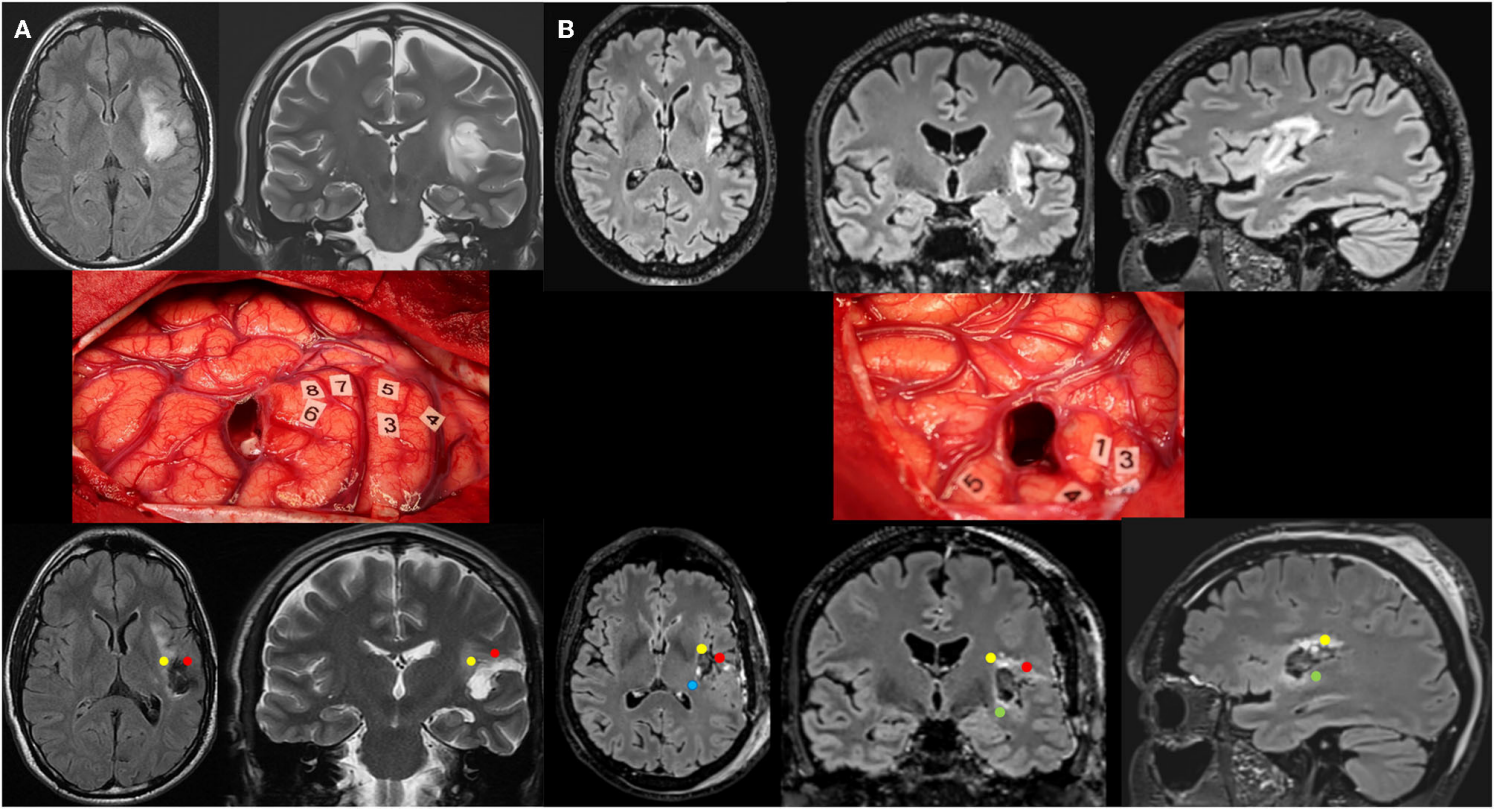
**(A)** Patient 1. Upper: Preoperative axial FLAIR (left) and coronal T2-weighted MRI (right) revealing a left posterosuperior insular LGG in a 35-year-old man who experienced seizures. Middle: Intraoperative view (the anterior part of the left hemisphere is on the right and its posterior part is on the left) after resection in awake patient, achieved up to eloquent structures, both at cortical and subcortical levels. Number tags show positive DES sites, i.e., primary motor cortex of the face (4), ventral premotor cortex eliciting anarthria when stimulated (3, 5), and primary somatosensory cortex of the face (6–8) within the lateral part of the retrocentral gyrus (rolandic operculum). According to this cortical mapping, a transopercular surgical approach has been selected via the alSMG (through the parietal operculum). In addition to the functional cortical areas, DES of white matter tracts allowed the detection of the critical subcortical neural networks (lSLF and AF). Lower: Postoperative axial FLAIR (left) and coronal T2 (right) MRI 3 months following surgery demonstrating NTR. The neurological examination was normal 3 months following surgery, after transitory phonological disorders. The diffuse WHO grade II oligodendroglioma was diagnosed, and no adjuvant treatment was administrated, with a regular surveillance. **(B)** Patient 2. Upper: Preoperative axial FLAIR (left), coronal FLAIR (middle), and sagittal FLAIR-weighted MRI (right) revealing a left posterosuperior insular LGG in a 59-year-old man who experienced seizures. Middle: Intraoperative view (the anterior part of the left hemisphere is on the right and its posterior part is on the left) after resection in awake patient, achieved up to eloquent structures, both at cortical and subcortical levels. Number tags show positive DES sites, i.e., primary motor cortex of the face (4), ventral premotor cortex eliciting anarthria when stimulated (1, 3), and primary somatosensory cortex of the face (5) within the retrocentral gyrus. According to this cortical mapping, a transopercular surgical approach has been selected via the lateral part of the retrocentral gyrus (through the posterior rolandic operculum). In addition to the functional cortical areas, DES of white matter tracts allowed the detection of the critical subcortical neural networks (lSLF, AF, IFOF, and somatosensory TCP). Lower: Postoperative axial FLAIR (left), coronal FLAIR (middle), and sagittal FLAIR-weighted MRI (right) demonstrating GTR. The neurological examination was normal 3 months following surgery. The diffuse WHO grade II astrocytoma was diagnosed, and no adjuvant treatment was administrated, with a regular surveillance. LGG, low-grade glioma; DES, direct electrical stimulation; alSMG, anterolateral part of the supramarginal gyrus; lSLF, lateral part of the superior longitudinal fasciculus (red circle); AF, arcuate fasciculus (yellow circle); IFOF, inferior fronto-occipital fasciculus (green circle); TCP, thalamocortical pathway (blue circle); GTR, gross total resection; NTR, near total resection.

**Figure 2:**
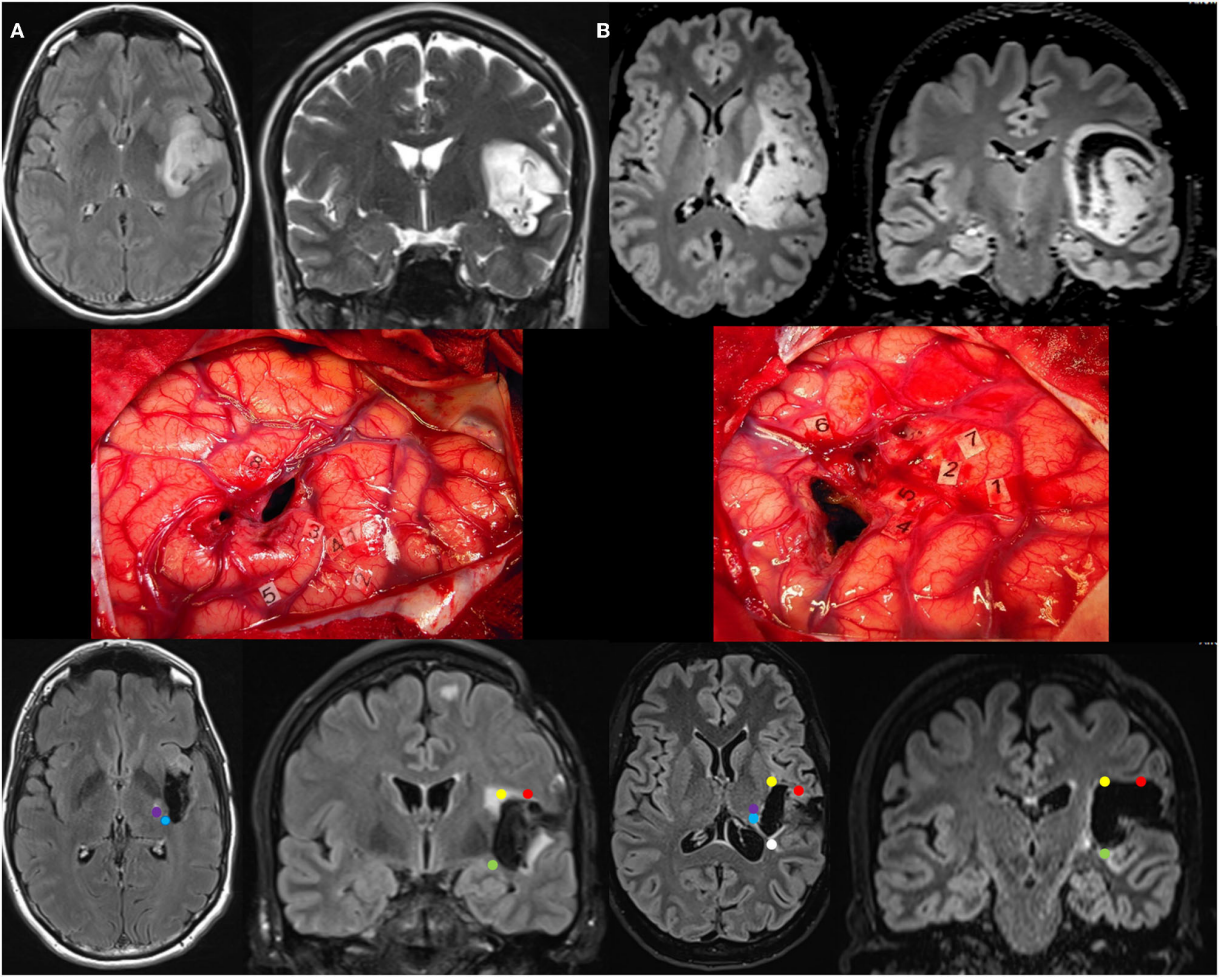
**(A)** Patient 3. Upper: Preoperative axial FLAIR (left) and coronal T2-weighted MRI (right) revealing a left posterosuperior insular LGG, incidentally discovered in a 42-year-old woman. Middle: Intraoperative view (the anterior part of the left hemisphere is on the right and its posterior part is on the left) after resection in awake patient, achieved up to eloquent structures, both at cortical and subcortical levels. Number tags show positive DES sites, i.e., primary motor cortex of the face (2), ventral premotor cortex eliciting anarthria when stimulated (1, 4), primary somatosensory cortex of the face (3) and upper limb (5) within the retrocentral gyrus, and superior temporal gyrus inducing anomia during DES (8). According to this cortical mapping, a transopercular surgical approach has been selected via the alSMG (through the parietal operculum) and the lateral part of the retrocentral gyrus. In addition to the functional cortical areas, DES of white matter tracts allowed the detection of the critical subcortical neural networks (lSLF, AF, IFOF, somatosensory TCP, PT, and OR). Lower: Postoperative axial FLAIR (left) and coronal T2 (right) MRI 3 months following surgery demonstrating NTR. The neurological examination was normal 3 months after surgery. A diffuse WHO grade II astrocytoma was diagnosed, and no adjuvant treatment was administrated, with a regular surveillance. **(B)** Patient 4. Upper: Preoperative axial FLAIR (left) and coronal T2-weighted MRI (right) revealing a voluminous left posterosuperior insular LGG in a 23-year-old woman who experienced seizures. Middle: Intraoperative view (the anterior part of the left hemisphere is on the right and its posterior part is on the left) after resection in awake patient, achieved up to eloquent structures, both at cortical and subcortical levels. Number tags show positive DES sites, i.e., primary motor cortex of the face (1), ventral premotor cortex eliciting anarthria when stimulated (2, 7), primary somatosensory cortex of the face (4, 5) within the retrocentral gyrus, and superior temporal gyrus inducing anomia during DES (6). According to this cortical mapping, a transopercular surgical approach has been selected via the alSMG (through the parietal operculum) and the lateral part of the retrocentral gyrus. In addition to the functional cortical areas, DES of white matter tracts allowed the detection of the critical subcortical neural networks (lSLF, IFOF, somatosensory TCP, and PT). Lower: Postoperative axial FLAIR (left) and coronal T2 (right) MRI 3 months following surgery demonstrating GTR. The neurological examination was normal 3 months after surgery. A diffuse WHO grade II astrocytoma was diagnosed, and no adjuvant treatment was administrated, with a regular surveillance. LGG, low-grade glioma; DES, direct electrical stimulation; alSMG, anterolateral part of the supramarginal gyrus; lSLF, lateral part of the superior longitudinal fasciculus (red circle); AF, arcuate fasciculus (yellow circle); IFOF, inferior fronto-occipital fasciculus (green circle); TCP, thalamocortical pathway (blue circle); PT, pyramidal tract (purple circle); OR, optic radiation (white circle); GTR, gross total resection; NTR, near total resection.

**Figure 3:**
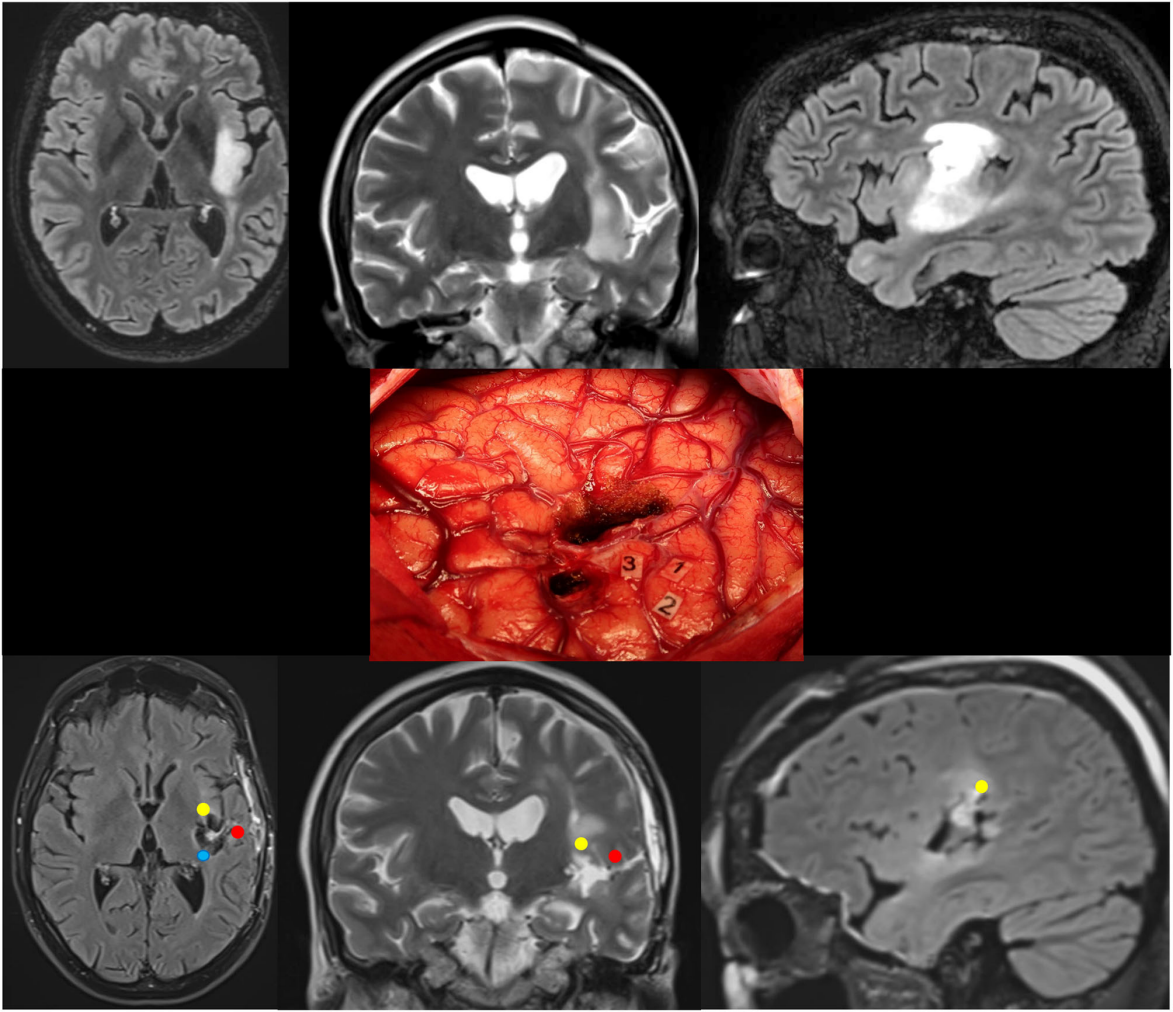
Patient 5. Upper: Preoperative axial FLAIR (left), coronal T2 (middle), and sagittal FLAIR-weighted MRI (right) revealing a left posterosuperior insular LGG in a 36-year-old woman who experienced seizures. Middle: Intraoperative view (the anterior part of the left hemisphere is on the right and its posterior part is on the left) after resection in awake patient, achieved up to eloquent structures, both at cortical and subcortical levels. Number tags show positive DES sites, i.e., ventral premotor cortex eliciting anarthria when stimulated (1, 2), and primary somatosensory cortex of the face (3) within the retrocentral gyrus. According to this cortical mapping, a transopercular surgical approach has been selected via the alSMG (through the parietal operculum) and the superior temporal gyrus. In addition to the functional cortical areas, DES of white matter tracts allowed the detection of the critical subcortical neural networks (lSLF, AF, and somatosensory TCP). Lower: Postoperative axial FLAIR (left), coronal T2 (middle), and sagittal FLAIR-weighted MRI (right) demonstrating NTR. The neurological examination was normal 3 months following surgery. A diffuse WHO grade II astrocytoma was diagnosed, and no adjuvant treatment was administrated, with a regular surveillance. LGG, low-grade glioma; DES, direct electrical stimulation; alSMG, anterolateral part of the supramarginal gyrus; lSLF, lateral part of the superior longitudinal fasciculus (red circle); AF, arcuate fasciculus (yellow circle); TCP, thalamocortical pathway (blue circle); NTR, near total resection.

Five patients were included in this study. Specific case details, with clinical, radiological, surgical, and pathological characteristics are shown in Table 1 and Figures 1–3. The mean age of operated patients was 39 years (23–59), with three males and two females. The five patients were right-handed. The tumor was diagnosed because of seizures in all cases but one (incidental discovery). There was no preoperative neurological deficit.

**Table 1:** Clinical, radiological, surgical and pathological characteristics.

	**Age/Sex**	**Clinical presentation**	**Intraoperative mapping**	**Surgical Approach**	**Pathology**	**EOR**	**Post-op Assessment**
Patient 1	35/M	Seizures	• Cortical mapping: speech arrest (vPMC), M1 (face), dysesthesia (face) (S1) • Subcortical mapping: Anarthria (lSLF), phonological paraphasia (AF)	Through alSMG	WHO Grade II Oligodendroglioma	NTR	• No epilepsy • No neurological deficit (transitory phonological disorders) • KPS 90
Patient 2	59/M	Seizures	• Cortical mapping: speech arrest (vPMC), M1 (face), dysesthesia (face) (S1) • Subcortical mapping: Anarthria (lSLF), phonological paraphasia (AF), semantics (IFOF), dysesthesia (TCP)	Through lRCG	WHO Grade II Astrocytoma	GTR	• No epilepsy • No neurological deficit • IK 100
Patient 3	42/F	Incidental discovery (tinnitus)	• Cortical mapping: speech arrest (vPMC), M1 (face), dysesthesia (face and upper limb) (S1), anomia (STG) • Subcortical mapping: Anarthria (lSLF), phonological paraphasia (AF), semantics (IFOF), dysesthesia/pain (TCP), involuntary movement (PT), visual disorders (OR)	Through alSMG + lRCG	WHO Grade II Astrocytoma	NTR	• Transient seizures then epilepsy control • No neurological deficit • KPS 100
Patient 4	23/F	Seizures	• Cortical mapping: speech arrest (vPMC), arrest of movement, M1 (face), dysesthesia (face) (S1), anomia (STG) • Subcortical mapping: Anarthria (lSLF), semantics (IFOF), dysesthesia (TCP), involuntary movement (PT)	Through alSMG + lRCG	WHO Grade II Astrocytoma	GTR	• No epilepsy • No neurological deficit (transitory right dysesthesia) • KPS 90
Patient 5	36/F	Seizures	• Cortical mapping: speech arrest (vPMC), dysesthesia (face) (S1) • Subcortical mapping: Anarthria (lSLF), phonological paraphasia (AF), dysesthesia/pain (TCP)	Through alSMG + STG	WHO Grade II Astrocytoma	NTR	• No epilepsy • No neurological deficit (transitory right dysesthesia) • KPS 90

*F, female; M, male; vPMC, ventral premotor cortex; S1, primary sensory cortex; M1, primary motor cortex; STG, superior temporal gyrus; alSMG, antero-lateral supramarginal gyrus; lRCG, lateral retrocentral gyrus; WHO, World Health Organization; NTR, near-total resection; GTR, Gross-total resection; KPS, Karnovsky Performance Status; lSLF, lateral superior longitudinal fasciculus; AF, arcuate fasciculus; IFOF, inferior fronto-occipital fasciculus; TCP, thalamo-cortical pathway; PT, pyramidal tract; OR, optic radiations*.

The cortical mapping was positive in the five patients, with articulatory/language and/or sensorimotor disturbances transitorily induced by DES. This enabled the selection of an optimal transcortical approach, *via* the alSMG (i.e., the anterior parietal operculum) in four patients and/or *via* the lRCG (i.e., the posterior rolandic operculum) in three cases. In one case, a combined approach was performed through the posterior part of the superior temporal gyrus in addition to the SMG. Moreover, the white matter tracts were identified by means of axonal DES in all cases, i.e., the lSLF before reaching the insula in the five cases, then the AF (four cases), the somatosensory TCP (four cases), the IFOF (three cases), the PT (two cases), and the OR in one case. Surgical resection was stopped into the contact of these functional pathways in the five patients.

Two patients had a gross total resection and three patients had a near-total resection on the postoperative FLAIR-weighted MRI. No permanent sensorimotor, language, or visual symptoms were experienced by any patient in this series. Histopathology results revealed the WHO grade II glioma (LGG) in all the cases (three astrocytomas and two oligodendrogliomas). No adjuvant medical treatment has been administrated in the postoperative period.

## Discussion

Despite the recent improvement of postoperative results following resection of insular gliomas (18), the rate of permanent deterioration remains high in series which still advocates the use of a trans-Sylvian approach, i.e., between 6 and 17% (19–22). In addition, among all the types of insular tumors, those involving the posterior part of the insula in the left hemisphere are more difficult to deal with (8), particularly when involving the zone II in the Berger-Sanai classification—thus resulting in a lower EOR (9, 22, 23). Indeed, its surgical approach is complex, because this area is deeper than the anterior insula. Moreover, since the bifurcation of the superficial Sylvian vein is frequently localized directly above the posterior insula, splitting the posterior part of the Sylvian fissure is more challenging. Finally, in the trans-Sylvian approach, the risk of spasm after skeletonizing the Sylvian artery is higher, which could have major consequences given the fact that the long perforating arteries, critical for the corona radiata, may arise from these posterior branches that overly the posterior insula (24).

To overcome these issues, the present case series detailed a transcortical approach to reach the left posterior LGG, thanks to optimized access through the lRCG and/or alSMG in awake patients. Indeed, this approach *via* the rolandic and/or parietal opercula offers a safe and effective corridor to the posterior insula, deeply located, by decreasing brain retraction by means of a large and straightforward working corridor, and by minimizing the vascular risks related to the opening of the Sylvian fissure. Here, no severe long-lasting worsening has been generated, in agreement with a low rate of morbidity (<6%) reported in previous surgical experiences which used a transcortical approach (5–7)—nonetheless by emphasizing in the present series the reproducibility of these results by using a transparietocentral opercular access regarding specifically LGG centered on the left posterior insula.

Furthermore, the favorable functional outcomes reported here may be attributed to the use of awake mapping, in line with recent series which evidenced a lower rate of persistent deficits in insular resection following awake procedure with DES compared with asleep craniotomy (4). Such a functional mapping is very important in this left posterior insular localization both for surgical access and concerning the limit of resection into the contact of the deep white matter tracts. In fact, conversely to the classical transcortical approach often reported through the frontal operculum in the literature, a more posterior approach *via* the left lRCG/alSMG implies not only to take account of the critical cortical sites mapped by DES but also to preserve the lSLF which is essential for articulatory processing (25). This corticocortical tract connects the junction between the posterior SMG and the posterior superior temporal gyrus with the ventral premotor cortex, and it runs more superficially than the insula (26). Because this auditory-motor pathway cannot be functionally compensated, since leading otherwise to permanent dysarthria when damaged (13), it should be imperatively identified by means of axonal DES as the upper functional limit of the transopercular approach to the left posterior insula. Of note, in the present series, the lSLF has been detected in all patients before reaching the insula.

A step forward, after removing the insular tumor, the eloquent fasciculi have also to be preserved within the deep white matter connectivity. Indeed, the region underneath the insula is a major cross-road, which includes several neural networks, that is, the AF, the somatosensory TCP, the PT, the OR, and the IFOF. The axonal fiber tract serving as the deep posterosuperior boundary is represented by the left AF, which arches around the posterior insula, situated more deeply than the lSLF, and which generates phonological paraphasia during DES (dorsal route) (11, 27). Moreover, the profound part of the posterior insula is very close to the posterior limb of the internal capsule. Therefore, the TCP which elicits dysesthesia or pain when stimulated and the PT which evokes movement disturbances during DES (25) should be identified and preserved in the depth of the surgical cavity. The OR may also be detected, causing visual disorders during DES and serving as the deep posterior limit of the resection (28). Finally, the left IFOF, which runs in the external capsule, can be identified by inducing semantic paraphasia during DES, thus serving as the lower and anterior boundary of the glioma removal (ventral route) (10, 11, 27). In the present series, one or several of these critical pathways have been mapped in all patients (Figures 1–3), avoiding to damage axonal fibers and generating long-lasting functional deterioration related to subcortical structural disconnections. Indeed, despite mechanisms of neural reconfiguration which may be elicited by slow-growing LGG (29), such a potential of neuroplasticity is very limited at the level of the white matter connectivity, thus with a high risk of permanent deficit in the event of tracts injury (16, 30, 31).

To sum up, a comprehensive investigation of the cortical and axonal functional anatomy by means of intraoperative DES should be considered for each patient with a left posterior insular glioma, both for selecting the best transopercular corridor and for optimizing the EOR while preserving the deep neural circuits.

## Conclusion

Although this is a small series, the present results support that a transcortical approach through the parietal and/or rolandic opercula (i.e., anterolateral part of the SMG and/or lateral part of the RCG) in awake patients represents safe and effective access to the left posterior insular LGG. In addition, detection and preservation of the functional connectivity using axonal stimulation mapping of the white matter bundles are needed in this major cross-road brain region to circumvent predictable long-lasting postoperative worsening.

## Data Availability Statement

The original contributions presented in the study are included in the article/supplementary material, further inquiries can be directed to the corresponding author/s.

## Ethics Statement

Ethical review and approval was not required for the study on human participants in accordance with the local legislation and institutional requirements. The patients/participants provided their written informed consent to participate in this study.

## Author Contributions

The author confirms being the sole contributor of this work and has approved it for publication.

## Conflict of Interest

The author declares that the research was conducted in the absence of any commercial or financial relationships that could be construed as a potential conflict of interest.

## Publisher's Note

All claims expressed in this article are solely those of the authors and do not necessarily represent those of their affiliated organizations, or those of the publisher, the editors and the reviewers. Any product that may be evaluated in this article, or claim that may be made by its manufacturer, is not guaranteed or endorsed by the publisher.
